# Effect of adapter duration on repetition suppression in inferior temporal cortex

**DOI:** 10.1038/s41598-017-03172-3

**Published:** 2017-06-09

**Authors:** Pradeep Kuravi, Rufin Vogels

**Affiliations:** Laboratorium voor Neuro- en Psychofysiologie, Department of Neurosciences, KULeuven, Leuven, Belgium

## Abstract

Many inferior temporal (IT) cortical neurons reduce their response when a stimulus is repeated. Proposed mechanisms underlying this repetition suppression range from “fatigue” to top-down expectations of repetition. Here we examine a prediction from simple fatigue-based models of adaptation: prolonging adapter duration will increase the adapter response, leading to more repetition suppression. To test this, we varied adapter duration from 300 to 3000 ms, keeping the test stimulus duration constant. We observed no effect of adapter duration on repetition suppression when averaging responses across the test presentation. This was not because of a ceiling effect, since repeated presentations of a short adapter increased repetition suppression. Examination of test stimulus responses showed increased repetition suppression with longer adapter durations during the initial response phase, which reversed at a later phase. Across neurons, we found that the degree of repetition suppression covaried with the ratio of the response during the initial transient and later sustained phase of the response during the long adapter presentation, suggesting overlapping mechanisms that underlie adaptation during the adapter and the delayed test. We propose a fatigue-based account in which fatigue increases non-linearly with adapter duration to explain these unexpected findings.

## Introduction

Most inferior temporal (IT) neurons decrease their response when repeating a visual stimulus^1–10^. The mechanisms underlying this repetition suppression are still poorly understood^11^. The proposed mechanisms range from firing-rate dependent fatigue and synaptic depression to top-down driven expectation suppression. So far, no evidence for top-down expectation-induced repetition suppression has been obtained in nonhuman primates^10, 12^. Hence, the most parsimonious explanation of repetition suppression in macaque IT currently relies on bottom-up or local fatigue mechanisms, such as firing rate dependent fatigue or reduced input due to synaptic depression or adapted afferents^6, 7, 11^.

**Figure 1:**
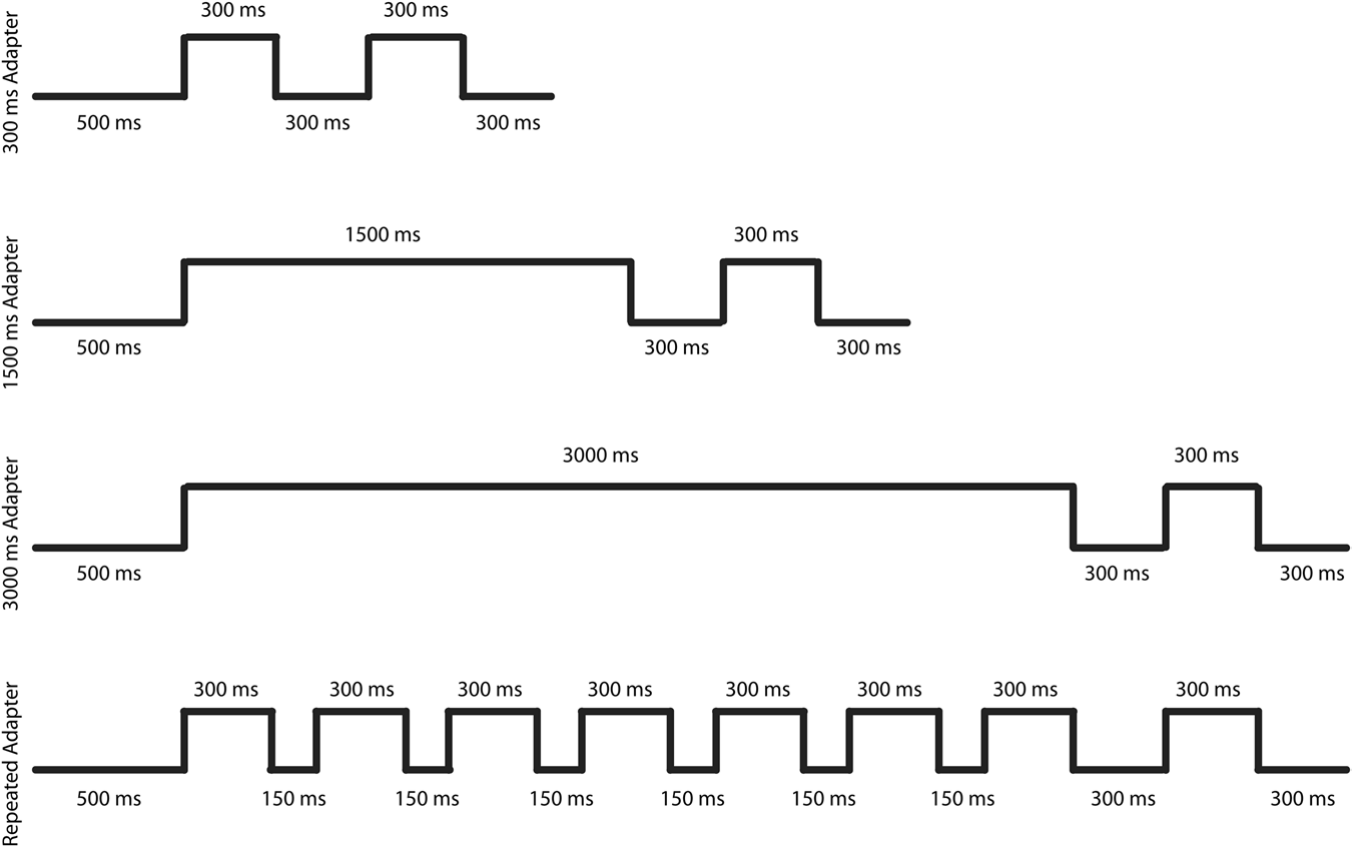
Adaptation protocols. The lower horizontal lines indicate periods during which only a fixation target was present, while the upper horizontal lines indicate stimulus presentations. Durations of the different periods are indicated below (for fixation target only periods) and above (for stimuli) the lines. The last 300 ms stimulus period correspond to the test stimulus presentation. Successful fixation during the entire trial resulted in a juice reward following the last fixation target-only period.

Simple fatigue-based models of repetition suppression predict that the degree of repetition suppression should depend on the duration of the adapter stimulus: the longer the adapter duration, the stronger the fatigue and resulting suppression. To the best of our knowledge and perhaps surprisingly, the effect of adapter duration has not been studied systematically and across a wide range of durations at the single unit level in awake IT cortex. To address this gap, we measured the responses of IT neurons to stimuli that followed adapters presented at different durations, ranging between 300 and 3000 ms (Fig. 1). Since the animals were obliged to fixate in a small window, we did not present adapter durations longer than 3000 ms. Irrespectively of adapter duration, the test stimuli had the same duration of 300 ms and followed the adapter stimulus offset after a fixed interstimulus interval (ISI) of 300 ms. The test and adapter stimuli could be either identical (repetition trials) or different (alternation trials). We also assessed whether the response suppression observed at different durations was inherited from previous visual areas with smaller receptive fields. To do this, we examined adaptation when the adapter and test stimuli were presented in different, non-overlapping positions in the visual field, similarly to De Baene and Vogels^6^.

## Methods

### Subjects

Two rhesus macaques (*Macaca mulatta*; male monkey D and female monkey K) served as subjects. Animal care and experiments were carried out in accordance with the national and European guidelines and were approved by the Animal Ethics Committee of the KU Leuven.

Details about implants and surgery can be found in Kaliukhovich and Vogels^10^ and will only be briefly summarized here. The placement of the plastic recording chamber was guided with a preoperative magnetic resonance imaging (MRI) scan and verified with MRI scans obtained postoperatively before and in-between recording sessions. For the latter MRI scans, the recording chamber, with the Crist grid located at the same position as during the recordings, was filled with a 1% solution of the gadolinium-based contrast agent Gadoteric acid (Dotarem). This combined with the insertion of tungsten wires, enclosed in glass capillaries, in the grid at 5 locations enabled visualization of the recording chamber, grid and electrode trajectories. Recording positions were estimated based on the MRI visualization of these markers combined with the microdrive depth readings of the white/gray matter transitions relative to the grid base.

Recordings were made from the lower bank of the rostral superior temporal sulcus of the right hemisphere. The anterior-posterior coordinates of the estimated recording positions ranged between 9 and 12 mm and between 15 and 16 mm anterior to the auditory meatus in monkeys D and K, respectively. The corresponding medial-lateral coordinates in those monkeys ranged between 24 and 25 mm, and 20 and 23 mm lateral to the midline.

### Recordings

Well-isolated extracellular action potentials of single IT neurons were recorded with tungsten microelectrodes. The electrode was lowered with a Narishige microdrive through a guide tube that was fixed in a Crist grid. The guide tube was grounded and served as a reference. Amplification and filtering was performed by a Plexon data acquisition system (Plexon Inc.). Recorded signals were preamplified with a headstage having an input impedance of >1GΩ. The signal was bandpass filtered between 250 to 8000 Hz for spikes and 0.7 and 170 Hz for LFPs. Action potentials from single cells were isolated online using the ‘time-window discrimination’ tool provided by the Plexon data acquisition system. Triggered spike-wave forms were saved at 40 kHz for later offline analysis (Offline Sorter; Plexon) in which single unit isolation was re-checked. Stable single unit recordings are expected to be biased towards large pyramidal cells. To overcome this bias, we recorded in some sessions also multi-unit activity by employing a low discrimination threshold level. Note that multi-unit activity may overrepresent the activity of interneurons since the latter have higher firing rates than pyramidal neurons and thus will contribute more to the multi-unit signal compared to a single unit signal. LFPs were recorded simultaneously with the same electrodes.

Eye position was measured online with an infrared-based eye tracking system (ISCAN EC-240A, ISCAN Inc.; 120 Hz sampling rate). The analog eye movement signal was saved with a sampling frequency of 1 kHz. In all tasks, we employed fixation windows that measured maximally 2° on a side. Eye positions, stimulus and behavioral events were recorded simultaneously with spiking activity and stored for later off-line analysis on a computer that was synchronized with the Plexon data acquisition system.

### Stimuli

We employed a stimulus set identical to that used in some of our previous studies on adaptation in macaque IT^7, 8^. The stimulus set consisted of 52 color images of 26 object classes (2 images per class) including human and monkey faces, human and monkey bodies, mammals, birds, fish, snakes, insects, trees, fruits, fractals and manmade objects. The size of the stimuli (maximum of horizontal and vertical dimensions of the bounding box) was approximately 5° of visual angle. The stimuli were presented on a uniform gray background on a CRT monitor (Phillips Brilliance 202P4, frame rate = 60 Hz, resolution = 1024 × 768 pixels) located 60 cm from the subject’s eyes.

### Search test

While advancing the microelectrode in IT, we searched for responsive neurons using a search test. On each trial of the search test, the monkeys were required to passively maintain their gaze on a red fixation target square (size = 0.17°) presented in the center of the monitor and visible during the entire trial. A trial started with the onset of the fixation target. After 300 ms of stable fixation, a stimulus was presented for 300 ms. To complete a trial and obtain a fluid reward, the monkeys had to maintain fixation for 300 ms poststimulus. The different images were presented foveally in a random manner. Once the spiking activity of a responsive neuron was well isolated or at a responsive multi-unit site, the search test was used to select two stimuli, with one of the two stimuli evoking a strong response (effective stimulus, labeled A) and the other little or no response (ineffective stimulus, labeled B). The two stimuli were selected on-line from the pool of 52 color images by inspection of post-stimulus time histograms (PSTH) of the responses of the neuron or multi-unit site to each of the stimuli.

### Adaptation tests

In a first series of experiments, we tested single units with the following adaptation test (Fig. 1). After onset of the fixation target, the monkey had to fixate that target for 500 ms. After that prestimulus fixation period, a stimulus was presented, which served as adapter. The adapter stimulus could be either the selected A or B stimulus and was presented for 300, 1500 or 3000 ms duration. The fixation target was presented on top of the centrally presented stimulus. After the presentation of the adapter stimulus, there was an interstimulus interval of 300 ms during which only the fixation target was presented. The interstimulus interval was followed by presentation of the test stimulus which lasted 300 ms. As for the adapter stimulus, the fixation target was presented on top of the test stimulus. The test stimulus was presented at the same central location as the adapter stimulus. The test stimulus was either A or B. After the offset of the test stimulus, the fixation target was presented for another 300 ms. Continuous fixation during the whole trial was rewarded with a liquid reward after the 300 ms post-test fixation period. After delivering the reward, we presented a full-field scrambled stimulus for 500 ms followed by a blank screen for 150 ms. Each scene was scrambled five times (20 scenes × 5 = 100 stimuli). During this period, the monkey was not required to fixate. The presentation of the scrambled stimulus served to reduce the carry-over of adaptation effects from the adapter and test stimuli to the subsequent trial. The median inter-trial interval was 1838 ms, with a minimum (percentile 0) of 1638 ms and a percentile 95 of 7112 ms. The 12 conditions (2 stimuli × 2 trial types (repetition (AA, BB) versus alternation (AB, BA)) × 3 adapter durations) of the adaptation test were presented in random order in blocks of 24 unaborted trials (i.e. each condition twice per block). The minimum number of unaborted trials per condition was 6, with a median of 10 (N = 92 neurons).

In the majority of recordings, we added 6 conditions in which during the period that the adapter stimulus would have been presented, only the fixation target was shown. Thus, these trials lasted for the same durations as those in which an adapter was presented but the test stimulus was preceded only by presentation of the fixation target (see Fig. 1). Thus, these 6 additional conditions consisted of presentations of A or B as test stimulus following one of the 3 durations (300, 1500 and 3000 ms) without presentation of the adapter. The timing was exactly the same as for the other corresponding 12 conditions. The 18 conditions (12 + 6) of the full adaptation test were presented in random order in blocks of 36 unaborted trials, with a minimum of 6 unaborted trials per condition.

In the next recording sessions, we tested an additional stimulus condition in which the adapter was shown repeatedly before presentation of the test stimulus (Fig. 1). After the prestimulus period, the adapter stimulus was presented 7 times for 300 ms each and the interstimulus interval between successive adapter presentations was 150 ms. After the 7^th^ presentation of the adapter stimulus, the interstimulus interval was 300 ms, as in the other adaptation conditions, and was followed by the presentation of the test stimulus. As before the adapter and test stimulus could be either A or B, with repetition and alternation trials. Thus, the repeated adapter conditions numbered 4 in total (2 stimuli × 2 trial types). Note that in these conditions the total duration of the adapter was 2100 ms, which was less than the longest stimulus duration of 3000 ms. The 4 repeated adapter conditions were presented randomly interleaved with the 300 and 3000 ms adapter duration conditions, yielding a total of 12 conditions. The 12 conditions were presented in random order in blocks of 24 unaborted trials, with a minimum number of 6 unaborted presentations per condition (median = 10 trials/condition). We tested both single units and multi-unit activity in different sessions.

In a separate set of recordings, we manipulated the stimulus position independently for the adapter and test stimuli: the adapter and test stimuli were presented at the same or a different, non-overlapping position. Each of the two stimuli could be shown at the 5° eccentricity in the lower or upper visual field. We employed two adapter durations: 300 and 3000 ms. Thus, this test included 32 conditions (2 positions × 2 stimulus types (adapter versus test) × 2 stimuli (A and B)) × 2 trial types × 2 adapter durations). The 32 conditions were presented in random order in blocks of 64 unaborted trials, with a minimum of 6 trials/condition (median = 10 trials/condition). We tested only single units in these recording sessions.

### Data analysis

For each recorded neuron and condition, we computed the mean firing rate to the adapter and test stimuli. Only unaborted trials were analyzed. Responses to the stimuli were computed within a 300 ms long analysis window starting at 60 ms after stimulus onset. In all experiments, the onset of the adapter and test stimuli were detected by a photodiode and those timings were employed to align the neural activity to the stimuli for the two stimulus types separately. For each neuron and condition we computed an adaptation contrast index:

$$\begin{array}{c}({\rm{n}}{\rm{e}}{\rm{t}}\,{\rm{r}}{\rm{e}}{\rm{s}}{\rm{p}}{\rm{o}}{\rm{n}}{\rm{s}}{\rm{e}}\,{\rm{t}}{\rm{o}}\,{\rm{a}}{\rm{d}}{\rm{a}}{\rm{p}}{\rm{t}}{\rm{e}}{\rm{r}}-{\rm{n}}{\rm{e}}{\rm{t}}\,{\rm{r}}{\rm{e}}{\rm{s}}{\rm{p}}{\rm{o}}{\rm{n}}{\rm{s}}{\rm{e}}\,{\rm{t}}{\rm{o}}\,{\rm{t}}{\rm{e}}{\rm{s}}{\rm{t}})/(|{\rm{n}}{\rm{e}}{\rm{t}}\,{\rm{r}}{\rm{e}}{\rm{s}}{\rm{p}}{\rm{o}}{\rm{n}}{\rm{s}}{\rm{e}}\,{\rm{t}}{\rm{o}}\,{\rm{a}}{\rm{d}}{\rm{a}}{\rm{p}}{\rm{t}}{\rm{e}}{\rm{r}}|+|{\rm{n}}{\rm{e}}{\rm{t}}\\ \quad {\rm{r}}{\rm{e}}{\rm{s}}{\rm{p}}{\rm{o}}{\rm{n}}{\rm{s}}{\rm{e}}\,{\rm{t}}{\rm{o}}\,{\rm{t}}{\rm{e}}{\rm{s}}{\rm{t}}|).\end{array}$$

The net responses were computed by subtracting the baseline firing rate from the firing rate in the 300 ms analysis window. The baseline activity was defined as the mean firing rate in a 100 ms long interval that ended at the onset of the adapter stimulus.

PSTHs were computed for each neuron and condition by averaging the net firing rate in bins of 20 ms, aligned on stimulus onset. Then, population PSTHs were created by averaging the normalized net firing rate across neurons per condition. Normalization was performed per neuron by dividing the net firing rate by the maximum firing rate in a 20 ms bin across the AA repetition and the BA alternation trials. The BB and AB trials were not further analyzed: these trials served only to make the test stimulus identity unpredictable to the monkey. We computed the significance of the difference between the population PSTHs of different conditions by a Wilcoxon signed rank test applied to the net firing rate in each of 20 ms bins from 60 ms to 300 ms after stimulus onset. The p values were corrected for multiple comparisons (bins) using the Benjamini and Hochberg^13^ False Discovery Rate method (q < 0.05 was considered as statistically significant). The standard errors of the mean responses in the population PSTHs were computed following the procedure by Loftus and Masson^14^ which removes the variance due to the differences in the overall mean response across neurons or sites.

LFPs were filtered offline with a digital 50-Hz notch filter (48–52 Hz fourth-order Butterworth FIR filter; Fieldtrip Toolbox, F.C. Donders Centre for Cognitive Neuroimaging, Nijmegen, The Netherlands) to remove 50 Hz. Trials in which the signal was <1% or >99% of the total input range were excluded. We employed the same method for spectral analysis of the LFP as De Baene and Vogels^6^ and Kaliukhovich and Vogels^10^. By convolving single-trial data using complex Morlet wavelets^15^ and taking the square of the convolution between the wavelet and signal, the time-varying power of the signal for every frequency was obtained. Averaging spectral maps (power as a function of frequency and time) across trials for a given condition and site produced a spectral map of that condition and site. The complex Morlet wavelets had a constant center frequency-spectral bandwidth ratio ${f}_{0}/{\sigma }_{f}$ of 7, with ${f}_{0}$ ranging from 15 to 170 Hz in steps of 1 Hz. The spectral maps of the sites were normalized at each frequency by the average power within the baseline window of 300 ms before adapter onset.

Since repetition suppression in IT LFPs is consistently present for only the high frequency power^6, 9^, we quantified the adaptation effects by pooling the normalized power for the gamma frequencies between 70 and 170 Hz. Adaptation contrast indices were computed using the thus averaged gamma power within a temporal window that ranged between 50 and 350 ms post stimulus onset.

## Results

**Figure 2:**
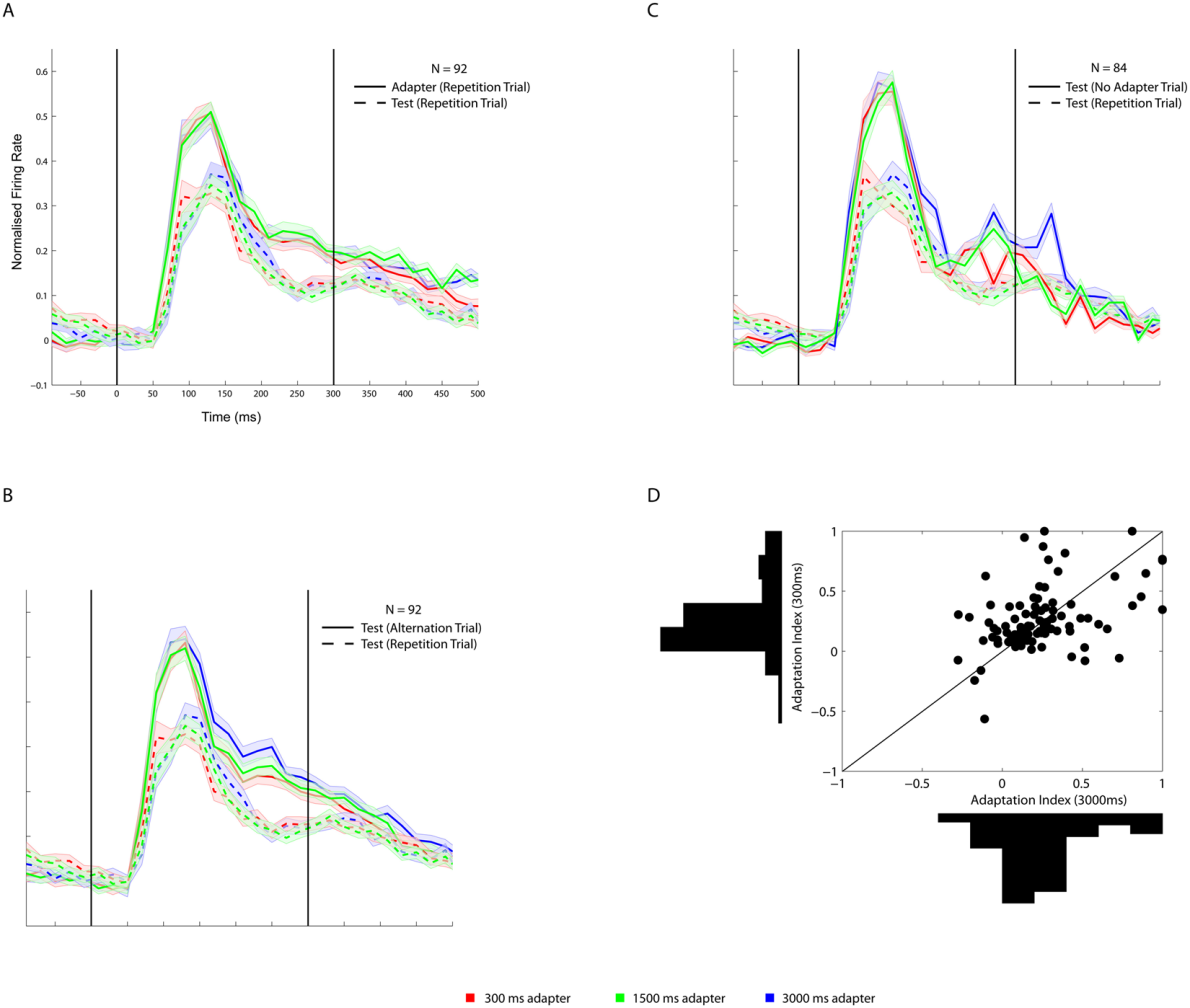
Responses to adapter and test stimuli as a function of adapter duration. (**A**) Normalized net firing rate to adapter and test stimuli in repetition trials for the 3 adapter durations. 0 corresponds to the onset of the adapter or test stimulus. The two vertical lines indicate the duration of the test stimulus and the adapter in the 300 ms duration condition. For the longer adapter durations, we show only the responses for the initial 500 ms of the stimulus presentation. (**B**) Normalized net firing rate to test stimuli in repetition and alternation trials for the 3 adapter duration conditions. Same neurons as in A. Same conventions as in A. (**C**) Normalized net responses to the same test stimuli without adapter (no adapter trial) or an adapter in repetition trials. The 84 neurons are a subsample of the 92 neurons in A and B. Same conventions as in A. Binwidth is 20 ms and no smoothing was applied. Shaded bands correspond to standard error of the mean. (**D**) Scatter plot and marginal distributions of adaptation contrast indices for 3000 ms (x-axis) and 300 ms (y-axis) adapter durations. The diagonal (equal x and y values) is indicated by a black line.

We measured the effect of adaptation to adapters of different duration (300, 1500 and 3000 ms; Fig. 1) of well-isolated single IT neurons in 2 rhesus monkeys. The adapter, irrespective of its duration, was followed by an interstimulus interval of 300 ms and a test stimulus that lasted 300 ms. Test and adapter stimuli could be either identical (repetition trials) or different (alternation trials). The two adapter/test stimuli were selected for each neuron, based on a preceding search test (see Methods). Figure 2 shows the mean normalized net responses to the adapter and test stimuli from 100 ms before to 500 ms after stimulus onset. Because the effects were similar in the two monkeys, we averaged the data of the animals. Note that each animal contributed a similar amount of neurons (monkey K: n = 44 out of a total of 92 neurons). As expected, the responses to the first 400 ms of the adapters was highly similar for the different adapter durations. Surprisingly, the adapter duration had only a slight effect on the response to the test stimulus in repetition trials (Fig. 2A and B; dashed curves). Computation of adaptation contrast indices (see Methods) using 300 ms analysis windows confirmed this: the median index was 0.21, 0.22 and 0.20 for the 300, 1500 and 3000 ms adapter duration conditions, which were all significantly greater than 0 (Wilcoxon median test; all p’s < 10^−10^). Statistical testing using pairwise Wilcoxon signed rank tests showed no significant differences between the adaptation contrast indices of the 3 adaptation duration conditions (see Fig. 2C for distributions and scatterplots of adaptation contrast indices of the 300 and 3000 ms adapter durations). As in previous studies^3, 7^, the adaptation effect was strongly stimulus specific, since the responses to the test stimuli in alternation trials were greater than those to the test stimuli in repetition trials (Fig. 2B), with little difference in response to the test stimuli in the alternation trials of the 3 adapter durations. A large majority of neurons (84 out of 92 neurons) were tested also without presenting an adapter, having the monkey fixate the fixation target during the same amount of time as in the adapter duration conditions before presenting the test stimuli. As expected, the responses in these no-adapter conditions were greater than those to the test stimuli in the repetition trials of the adaptation conditions (Fig. 2C) and similar to the test stimuli in the alternation trials. Thus, the reduced responses to the repeated stimulus in the adaptation conditions were driven by the adapter stimulus and were adapter-identity specific.

**Figure 3:**
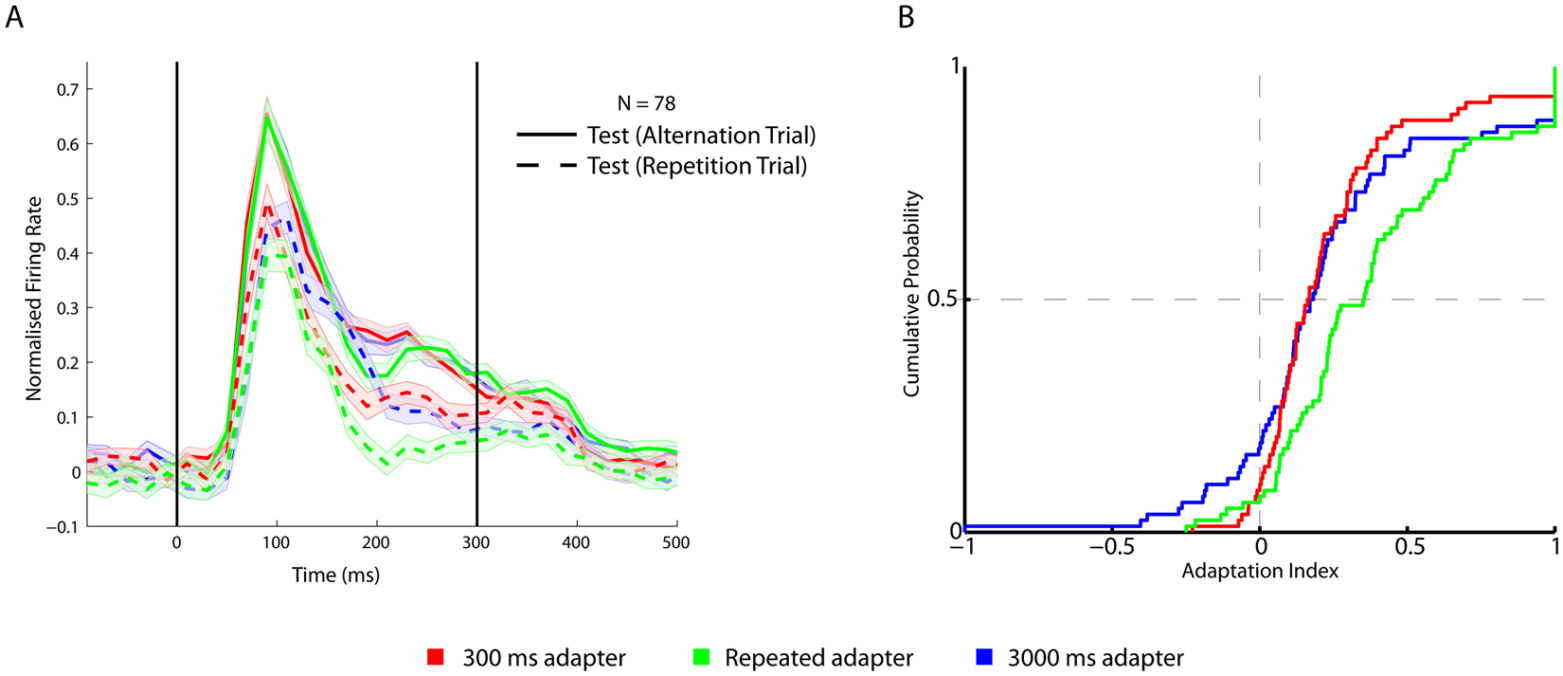
Responses following short, long and repeated adapter. (**A**) Normalized net firing rate to test stimuli following a 300 ms, 3000 ms and the repeated adapter in repetition and alternation trials. The firing rates for single units and multi-unit sites were averaged. Same conventions as in Fig. 2. (**B**) Cumulative distributions of the adaptation contrast indices for the 300 ms adapter duration, 3000ms adapter duration and repeated adapter conditions.

The absence of an adapter duration effect when considering the 300 ms analysis window appears to be in contrast to previous studies that showed a marked increased suppression of the response with repeated presentations of the adapter stimulus^3, 8^. However, increasing adapter duration is quite different from increasing the number of repetitions, despite the fact that the total exposure to the adapter stimulus increases in both manipulations. Repeating an adapter stimulus produces a transient response peak with each repeated adapter, while such a response peak is present only at the onset of the prolonged adapter. This transient response peak to the adapter might be a strong determinant of repetition suppression. Furthermore, constant stimulation might cause adaptation of the response to the prolonged adapter, thus weakening its effect on the later test stimulus. In other words, a repeated adapter, with its stimulus on- and offsets, might cause stronger adaptation than a long, continuous presentation of the adapter. To verify this directly, we tested both single and multiunits with a 300 ms and 3000 ms long continuous adapter stimulation and with a repeated 300 ms adapter (Fig. 1). The latter repeated adapter condition consisted of 7 presentations of the 300 ms adapter, with an interstimulus interval (ISI) of 150 ms. Note that the total exposure to the adapter was less in the repeated adaptation condition, lasting only 2100 ms, than in the 3000 ms adapter duration condition. Despite the shorter exposure, the repeated adapter condition produced a stronger stimulus selective repetition suppression than the 3000 ms adapter duration condition (Fig. 3A; single and multiunit activity was averaged since these showed similar effects). The median adaptation contrast index was 0.35 for the repeated adapter condition (Fig. 3B), which was significantly higher than those for the 300 (median index = 0.16; Wilcoxon signed rank test: p < 10^−6^; n = 78) and 3000 ms adapter duration conditions (median = 0.18; Wilcoxon signed rank test: p < 10^−5^).

**Figure 4:**
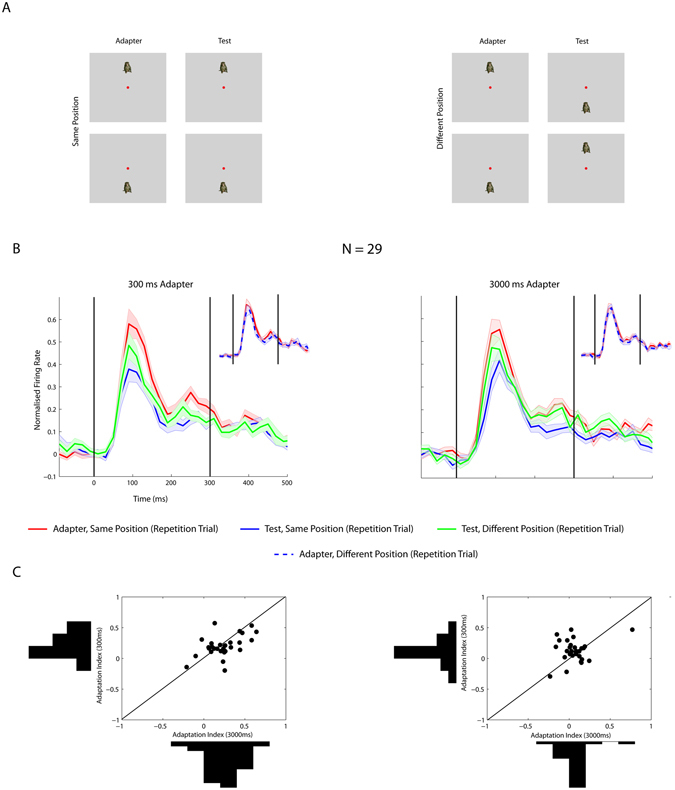
Repetition suppression when adapter and test stimuli were presented at the same or a different position. (**A**) Illustration of stimulus conditions. Adapter and test stimuli were presented at the same (left) or different positions above or below the fixation target along the vertical meridian. (**B**) Normalized firing rate of single IT units to adapter and test stimuli when the adapter and test stimuli were presented at the same position or at a different position during repetition trials. Left: adapter duration equaled 300 ms; right: adapter duration equaled 3000 ms. The insets compare the firing rates to the same adapter stimuli when followed by a test stimulus at the same or a different position. Same conventions as in Fig. 2. (**C**) Scatter plot and marginal distributions of adaptation contrast indices for 3000 ms (x-axis) and 300 ms (y axis) adapter durations. Left: Adapter and test presented at the same position; right: adapter and test presented at different positions. The diagonal (equal x and y values) is indicated by a black line.

It is unclear to what degree the repetition suppression we observed in IT is inherited from earlier stages. Furthermore, the inheritance of adaptation effects from earlier areas may well depend on adapter duration. For instance, it is possible that the contribution of adaptation at earlier levels to the effects seen in IT increases with stimulus duration. To assess this, we presented the adapter and test stimuli either at the same or a different retinal position (Fig. 4A). Stimuli were presented at 5° eccentricity in either the upper or lower visual field. When the adapter and test stimuli are presented at different positions, the observed repetition suppression cannot be due to inheritance from earlier areas with small receptive fields, such as V1, V2 and likely V4. Figure 4B plots the averaged normalized net single unit responses for the adapter and for the test stimuli at the same (blue curve) and a different position (green curve) as the adapter stimulus. For both adapter durations, the median adaptation contrast indices were significantly larger for the same compared with the different position conditions (Wilcoxon signed rank test; 300 ms: p < 0.0005; 3000 ms: p < 0.005), but repetition suppression was still present for both durations when the position of the adapter and test stimuli differed by 10°. Indeed, adaptation contrast indices were significantly greater than 0 for both the same and different position conditions for both the 300 ms (adaptation contrast indices: same position: 0.18; different position: 0.12; Wilcoxon median tests: both p’s < 0.005) and 3000 ms adapter durations (same position: 0.24; different position: 0.05; both p’s < 0.05). The median adaptation contrast indices were statistically indistinguishable for the 3000 compared with the 300 ms duration for both the same and different position conditions (Wilcoxon signed rank tests; both p’s > 0.19; Fig. 4C). Thus, adapter duration had negligible effects on the overall responses, irrespectively of the relative positions of the adapter and test stimuli, excluding a strong contribution of inheritance of adaptation at previous stages to the (absent) overall duration effect.

**Figure 5:**
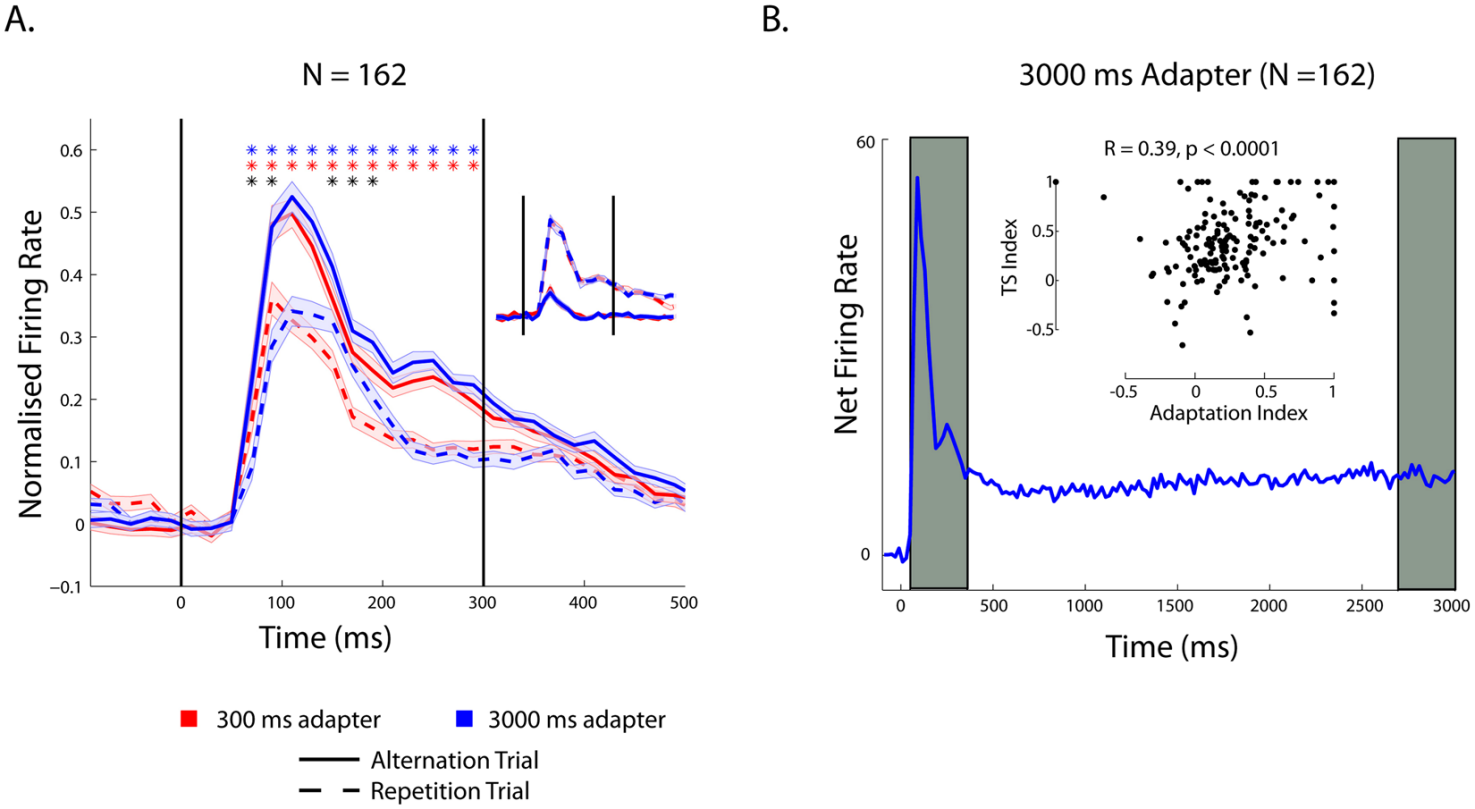
Time course of responses to test and adapter stimuli. (**A**) Normalized firing rate to test stimuli following the 300 and 3000 ms adapter in repetition and alternation trials. Average of all recorded single units. The inset shows the responses to the adapter stimuli in the same repetition and alternation trials for the first 500 ms of the two duration conditions. The blue and red stars indicate significant differences between the alternation and repetition trial test responses for the 3000 ms and 300 ms duration condition, respectively. The black stars indicate significant differences between the 300 and 3000 ms test stimulus responses in repetition trials. No differences between the two duration conditions were significant for the alternation trial test responses. Significant differences: Wilcoxon signed rank test: q < 0.05, corrected for multiple comparisons. Same conventions as in Fig. 2. (**B**) Mean net firing rate during the 3000 ms adapter, averaged across all single units. The shaded bars indicate the time windows employed to compute the TS index. Inset panel: scatter plot of TS index versus adaptation index. A positive TS index corresponds to a relatively weaker sustained response.

Closer examination of the responses for the different adapter durations in Figs 2, 3 and 4, which represent independent neuronal samples, shows a consistently different time course of the response to the test stimuli in repetition trials amongst the three adapter durations, despite similar average responses. This can be seen clearly when pooling all the available single unit data for the 300 and 3000 ms adapter durations of the (same position) repetition and alternation trials (Fig. 5A). Although the peak responses to the test stimuli following the different adapter durations in repetition trials were indistinguishable, there was a significantly stronger initial test response for the 300 compared to the 3000 ms adapter duration followed by a significantly more sustained, stronger response for the longer adapter duration condition. The significances shown in Fig. 5A were based on false discovery rate corrected Wilcoxon signed rank tests using a bin width of 20 ms. There was no significant late enhancement of the response to the test stimuli in alternation trials for the 3000 compared to the 300 ms adapter durations and also no difference between the response onset time course for the two duration conditions in the alternation trials. Thus, the difference between the time courses of the test responses for the two adapter duration conditions were not related to the timing of the stimuli per se, but were specific to stimulus repetition. Note that in this larger sample of neurons (n = 162), median adaptation indices, computed using a 300 ms analysis window, were again similar and statistically indistinguishable for the 300 (median = 0.20) and 3000 ms long adapter durations (median = 0.21; Wilcoxon signed rank test: p = 0.11).

**Figure 6:**
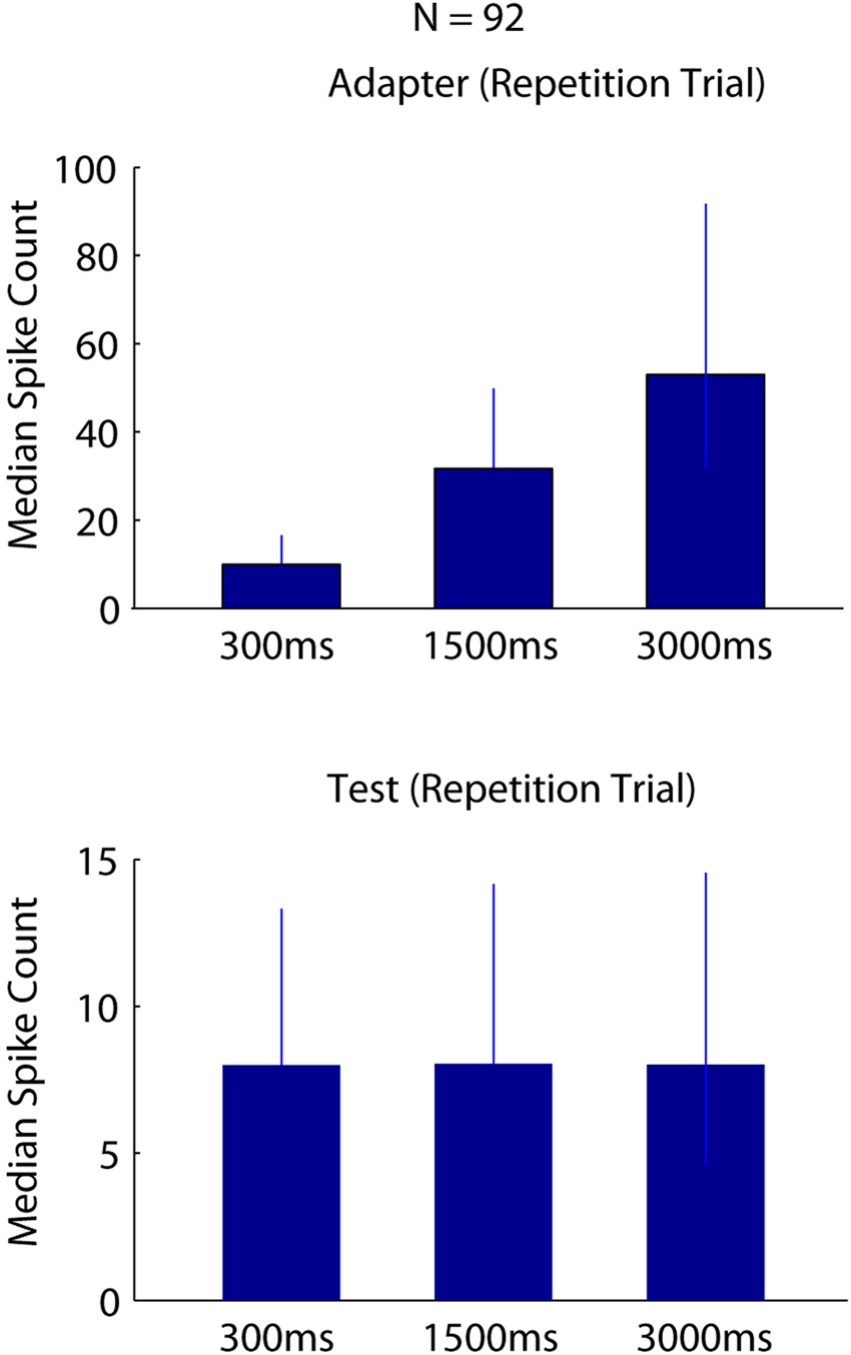
Integrated spike counts of single IT units during adapter and test presentations for different adapter duration conditions compared. (**A**) Median (and quartiles) spike counts integrated across the whole adapter presentation for 300, 1500 and 3000 ms adapter durations. (**B**) Median spike counts during the whole 300 ms long test presentation following the 300, 1500 and 3000 ms long adapter. Data from repetition trials only.

These data show that adaptation duration has a peculiar effect on the time course of the response to the repeated stimulus without affecting the overall spike count in a window that encompasses the test duration. The absence of an overall effect of adapter duration on overall spike count can be seen also when computing the median number of spikes during the test stimulus presentation for all 92 single neurons that were tested with 3 adapter durations (Fig. 6, bottom). These highly similar spike counts during the test stimulus presentations are in strong contrast with the increasingly higher spike counts, integrated during the adapter stimulus presentation, with increasing adapter duration (Fig. 6, top). Thus, the fivefold increase in spike counts obtained by prolonging the adapter presentation had no effect on the overall spike count computed during the test presentation (compare top and bottom panels of Fig. 6).

As shown in Fig. 5B, the response of the population of single IT neurons showed a transient increase in response shortly after adapter onset followed by a lower sustained response during the later part of the adapter. One potential factor that may influence the size of the repetition suppression in the long adapter duration condition is the activity in the sustained part of the response, just before the ISI between the adapter and the test stimulus. To test this, we took as measure of the sustained response strength of a neuron the non-normalized mean firing rate computed during the last 300 ms of the 3000 ms adaptation duration. If the size of the repetition suppression depends on the late sustained response to the adapter, one would predict a positive correlation between the degree of repetition suppression (adaptation contrast index) and the sustained response strength. Contrary to this prediction, we observed a statistically significant negative correlation (Spearman R = −0.37; p < 0.00001; n = 126 single neurons) between the adaptation contrast index and the thus computed sustained response strength. Hence, the degree of adaptation decreased with increasing sustained response strength. This also held when computing the correlation between the adaptation contrast index over the first 100 ms of the response to the stimuli (analysis window: 60–160 ms) and the sustained response strength (analysis window for adapter stimulus: 2700–3000 ms; R = −0.19; p < 0.05).

One possible explanation of this negative correlation between the sustained response strength and repetition suppression is that neurons with a high sustained response adapt less, both during the course of the response to the adapter as when comparing adapter and test responses (repetition suppression). To examine this, we computed for each single neuron tested in the 3000 ms adapter duration condition a transient-sustained (TS) index that contrasted the net firing rate T in a 60–360 ms window, which captures the transient phase of the adapter response, with the firing rate S in a 2700–3000 ms window, which measures the late sustained part of the response: TS = (T − S)/(|T| + |S|). The more adaptation during the course of the response to the long adapter, the higher the TS index. We observed a positive correlation between the adaptation contrast index and the TS index for the 3000 ms adapter duration condition (inset in Fig. 5B; Spearman rank correlation R = 0.39, p < 0.0001; n = 162 single neurons). This positive correlation was not a spurious result of having the same firing rates for the first 300 ms long window in both the adaptation and TS index. Firstly, the partial Spearman correlation with the firing rate in the first 300 ms window as covariate was also positive and statistically significant (partial R = 0.41; p < 0.0001). Secondly, when using an alternative adaptation contrast index that contrasts the test stimulus in alternation trials (instead of the response to the adapter in the first 300 ms window) with the test stimulus in repetition trials, the correlation between that adaptation index and the TS index remained significantly positive (Spearman R = 0.31; p < 0.0001).

**Figure 7:**
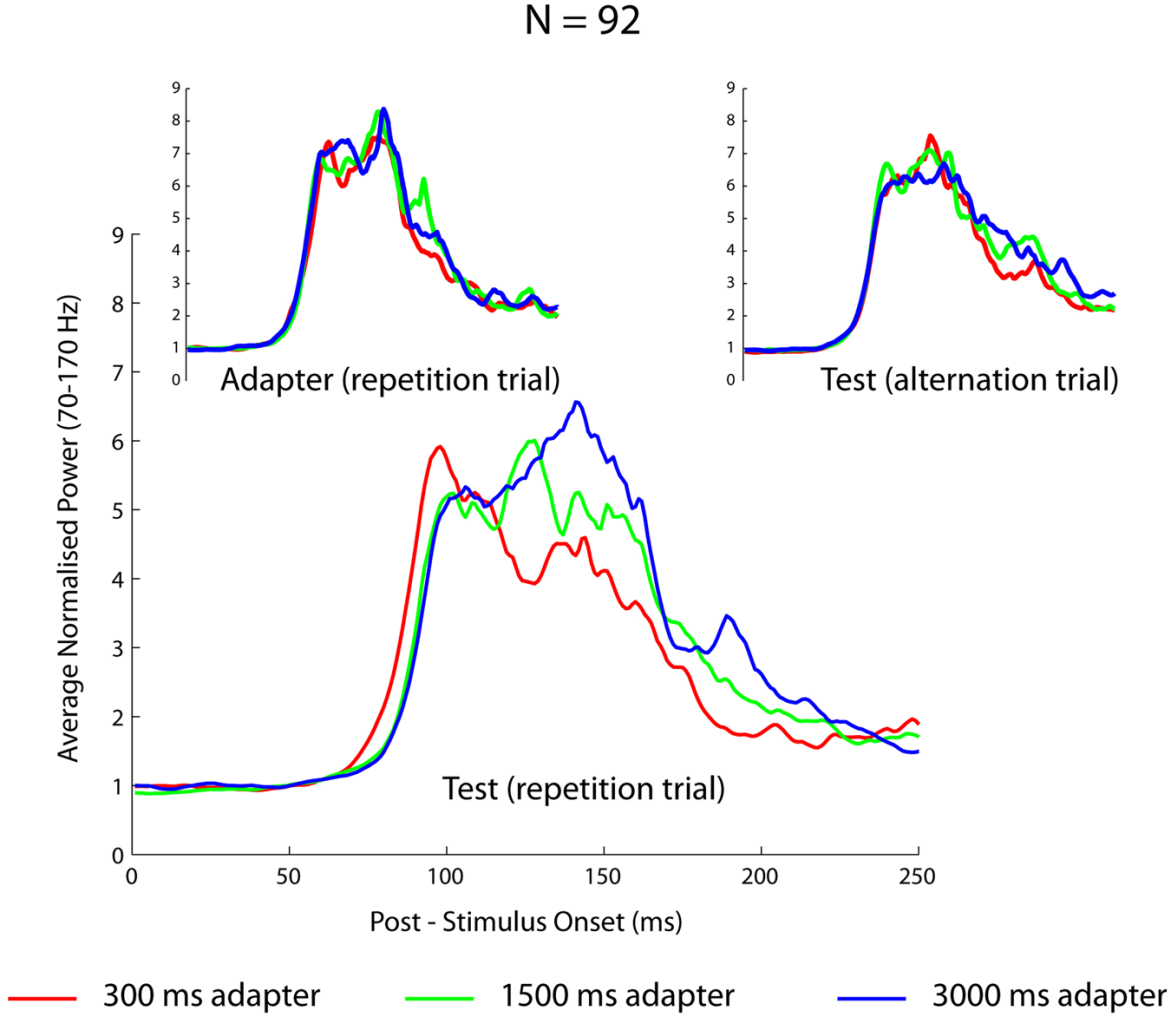
Gamma power (70–170 Hz) to adapter and test stimuli as a function of adapter duration. Averaged normalized gamma power during the first 250 ms of stimulus presentation for the test stimuli following a 300, 1500 and 3000 ms adapter in repetition trials. Insets shows averaged normalized power for the adapter in the same repetition trials (left inset) and test stimuli in alternation trials for the 3 different adapter duration conditions. The LFPs were obtained simultaneously with the single units of Figs 2 and 6.

Together with spiking activity, we measured also local field potentials. Time frequency analysis using Morlet wavelets (see Methods) of the LFPs showed evidence of repetition suppression for frequencies above 70 Hz in both animals. Figure 7 shows the average normalized power in the 70–170 Hz frequency band for the adapter and test stimuli in repetition and alternation trials and this for the 3 adapter duration conditions separately. The average high-frequency band power, measured in a window of 60 to 300 ms post-stimulus onset, was smaller during the test presentations in repetition trials, compared with the adapter, and this held true for each adapter duration (Wilcoxon sign rank tests; all P’s < 10^−9^; N = 92 sites). As for the spiking activity, the power to the repeated test stimulus increased faster after the 300 ms compared with the longer adapter durations. Thus, the average power to the repeated test stimulus, measured in a window from 60 to 100 ms post-stimulus onset, was significantly greater for the 300 ms compared to the 3000 ms adapter duration (Wilcoxon signed rank test; P < 0.0005). However, there was greater power with increasing adapter durations in the later window that ranged from 140 to 200 ms post-stimulus onset (difference 300 versus 3000 adapter duration: Wilcoxon signed rank test; P < 10^−6^). Note that these early and late analysis windows correspond to the time at which the spiking activity showed significant differences between the 300 and 3000 ms (Fig. 5A).

## Discussion

We expected that increasing the duration of an adapter stimulus would increase the degree of repetition suppression of the spiking activity in macaque IT. However, we found that large variations in adapter duration (in the range of 300–3000 ms) did not affect the overall degree of repetition suppression. This held true when adapter and test stimuli were presented at identical or different visual field positions. The invariance of overall repetition suppression with adapter duration did not result from a ceiling effect, since the repeated presentation of a short adapter stimulus produced stronger repetition suppression. An analysis of the time course of the spiking activity and the LFP gamma power showed changes in the response to the repeated stimulus with adapter duration: the initial part of the response to the repeated stimulus was greater for shorter compared to longer adapter durations, while the opposite was true for the later part of the response. These changes in time course of the test response were consistent across different independent experiments. Furthermore, we found that IT neurons that responded in a transient manner to a long duration adapter stimulus also showed stronger repetition suppression.

Previous studies of awake macaque IT repetition suppression varied adapter durations either within a smaller range and across neurons^3^ or for shorter durations^5^. Sawamura and coworker^3^ tested partially overlapping sets of neurons for durations varying between 300 and 900 ms and observed similar repetition suppression for these durations. Although this finding agrees with that of the present study, the absence of an effect of adapter duration in that study needs to be interpreted with caution since comparisons were made across small only partially overlapping samples of neurons. Also, the adapter duration range was quite low. Liu *et al*.^5^ found also similar time courses and degree of repetition suppression for a 150 and 500 ms adapter duration, tested in different neuronal samples. However, the degree of repetition suppression was significantly smaller for a short 80 ms adapter compared to the 500 ms duration. Combined with the result of the present study, this suggests that very short adapter durations produce less repetition suppression than longer ones, but this overall increase in repetition suppression with adapter duration appears to be limited to durations shorter than 150 ms (according to)^5^ or 300 ms (present study).

Patterson and coworkers^16^ varied adapter duration, ranging between 400 ms and 40 s, in anesthetized macaque V1. An adapter duration of 4 s showed stronger adaptation effects than a 0.4 s duration for small stimuli but not for stimuli that were much larger than the receptive field. The effect of adapter duration was mainly present in the later part (200–400 ms after stimulus onset) of the response and virtually absent in the initial part. Also, an ISI of 100 ms abolished repetition suppression in V1 for a 400 ms adapter duration, while it was still present at a 1 s ISI for 4000 ms adapter duration. This contrasts with repetition suppression in IT. First, repetition suppression in IT is present for short adapter durations with an ISI longer than 100 ms (ref. 3; present data). Second, there is no overall effect of adapter duration in IT between 300 and 3000 ms for stimuli that are very likely not larger than their receptive field, and third, the time courses of adaptation effects in V1 as a function of adapter duration are very different from those seen in IT. While this may reflect genuine differences between adaptation effects in V1 and IT, it is possible that the different effects of adapter durations in two studies reflect the different nature of the adapter stimuli. Patterson and coworkers employed drifting gratings as stimuli, while we employed static stimuli. Drifting gratings produce a more sustained stimulation than a static stimulus. In fact, a drifting grating may be more comparable to our repeated adapter condition, which also produced stronger adaptation than the static stimulation conditions.

To our knowledge, surprisingly few human imaging studies examined the effect of adapter duration on repetition suppression. Fang and coworkers^17^ compared viewpoint-specific fMRI adaptation in human subjects following a rather long +25 s exposure to a single viewpoint of a face with a short 400 ms exposure to that viewpoint. They found a stronger release of adaptation in FFA when the viewpoint between adapter and test stimulus differed by 90° but not by 30° for the long compared with the short duration adaptation. This was not the case in LO, despite the presence of viewpoint-specific adaptation in this area. Adaptation in the STS was found for the long but not the short duration adapter. These results suggest different adaptation effects for these two extreme durations, which might be area-dependent. However, the face stimulus in the long but not short adaptation condition was drifting during the exposure, which makes a clean comparison between adapter durations and between our monkey IT and their human study difficult.

Zago and coworkers^18^ varied systematically exposure durations, ranging between 40 and 1900 ms, in a fMRI repetition priming paradigm. They found that repetition suppression in occipito-temporal cortex peaked with a prime exposure duration of 250 ms. They found no difference between 350 and 1900 exposure durations. Although these findings agree with our study (and that of Liu *et al*.^5^ observation of less repetition suppression for a 80 ms adapter), the studies are difficult to compare because of the highly different paradigms: we employed a short 300 ms ISI with no intervening stimuli while in Zago *et al*.’s^18^ priming paradigm intervening stimuli (including a mask following the stimulus) between repetitions were present^18^, leading to variable and long intervals between the first and second presentation of the same stimulus (>2 s up to minutes).

A recent human ERP study examined the effect of adapter duration, ranging between 200 to 5000 ms, on the response evoked by the repeated presentation of faces^19^. Their design, using static adapters and a short 500 ms ISI, was comparable to ours. Since for most of our neurons, the two adapter stimuli belonged to different categories, the scrambled face versus face adapter comparison (their “generic” adaptation)^19^ comes the closest to our stimulus conditions. Zimmer and coworkers^19^ reported that the latter stimulus specific adaptation increased with adapter duration for the early P100 and the late P2 ERP components but less clearly for the N170 and the late N250 components. The current source(s) in the brain of these different ERP components are uncertain which makes a comparison with our IT data difficult. Nonetheless, it is tempting to speculate that the duration effect of the P100 component is related to the stronger adaptation effect with increasing adapter duration at 60–100 ms after stimulus onset in monkey IT. However, the later reversal of the adapter duration effect that we observed does not appear to be present in these human ERP data^19^.

In auditory cortex, longer electrical stimulation increases afterhyperpolarization, which underlies at least to some degree adaptation in that cortex^20^. Apart from firing rate dependent afterhyperpolarization, synaptic depression has also been implicated in causing adaptation. Synaptic depression is mainly due to presynaptic mechanisms such as synaptic depletion (for reviews see refs 21, 22) that depend on presynaptic activity. Thus, it is reasonable to suppose that both post-synaptic afterhyperpolarization and presynaptic synaptic depression increases with stimulus duration. However, both mechanisms, together with short-term spike rate adaptation^23^, will also induce adaptation during the stimulus presentation, especially during long adapter durations. This may explain why IT neurons show a transient increase in the response followed by a lower sustained period of activity. Interestingly, we found that the ratio between the transient and late sustained part of the response correlated with the degree of repetition suppression. A similar finding has been obtained in area MT^24, 25^ for brief duration (65ms) motion stimuli presented with short interstimulus intervals (32 ms). Furthermore, the IT neurons with a higher response strength during the late sustained phase of the adapter response showed less repetition suppression. Both findings can be explained by assuming that at least some mechanisms are common to the adaptation occurring during the adapter presentation and the adaptation to the test stimulus that is presented after a 300 ms ISI. It is possible that for instance the bulk of synaptic depression occurs during the initial part of the response – the transient phase – and then remains present during the continuous stimulation. In other words, the assumption here is that the degree of fatigue/depression depends in a strong nonlinear way on adapter duration. During the subsequent ISI there is only a partial recovery of the synaptic depression, resulting in repetition suppression for the test stimulus. We observed a stronger suppression for the long compared with the short adapter duration at the initial phase of the response to the test stimulus. Within the above scheme, this would imply that long duration stimulation will produce somewhat stronger fatigue (or less recovery, as observed in V1)^16^ than the short duration. The smaller degree of repetition suppression for the long compared to the short adapter duration in the later part of the test response might then result from the faster and initially stronger response to the test stimulus following a short duration adapter, resulting in relatively more adaptation during the later part of the test stimulus presentation for the short duration. In other words, we hypothesize that because there is a smaller transient response for the test stimulus in the 3000 ms duration condition – relative to the 300 ms duration condition –, there is less effect of spike-rate dependent adaptation during the later course of the response in the 3000 ms test condition, which manifests itself as a relatively greater late test response – again, relative to the 300 ms condition-. Part of the suppression may occur already upstream from IT, which can explain why overlapping adapter and test presentations produce stronger repetition suppression than non-overlapping presentations. Finally, it should not be forgotten that repetition suppression observed at the level of a single neuron also reflects network activity^7^. Thus, recurrent connections between active neurons within IT will contribute to the suppression effects seen in a single neuron, especially during the later parts of the response.

The above qualitative framework assumes that repetition suppression results from fatigue mechanisms like response-driven fatigue and/or synaptic or input fatigue^6, 11^. However, it has been proposed that repetition suppression reflects top-down expectations about stimulus repetition^26^. Although evidence for this proposal is lacking at the single unit level in IT^10^, it could explain why the overall level of repetition suppression is similar for different adapter durations since expectations about repetition are unlikely to depend on stimulus duration. Note that to avoid that our adaptation protocol induced expectations of repetition we had an equal number of repetition and alternation trials. Thus, when repetition suppression reflected a fulfilled expectation of repetition, as postulated by Summerfield and coworkers^26^ then this expectation must be a rather fixed “prior” that is not malleable to changes in repetition probability (which was 0.50 in our paradigm). Furthermore, as far as we know, such a predictive coding account of repetition suppression does not predict the differential effects of adapter duration on repetition suppression during the course of the response that we observed in the present study.

To summarize, we found that increasing the duration of a static adapter beyond 300 ms and up to 3000 ms does not produce an overall increase of the degree of repetition suppression, despite the increase in the number of spikes during the adaptation period. However, prolonging adapter duration resulted in an increased repetition suppression during the initial phase of the response but a decreased repetition suppression during the later phase of the response to the test stimulus. The degree of repetition suppression for the test stimulus correlated with the relative degree of reduction of the sustained phase of the response, suggesting at least overlapping mechanisms that drive the response during the adapter duration and the subsequent repetition suppression to a delayed test stimulus.

### Data availability

The data and analysis code that support the findings of this study are available from the authors upon reasonable request.
